# An additively manufactured titanium tilting suture anchor: a biomechanical assessment on human and ovine bone specimens

**DOI:** 10.3389/fsurg.2023.1195728

**Published:** 2023-11-30

**Authors:** Ali Abedi, Farzad Pourghazi, Maysa Eslami, Mohammad Hossein Nabian, Ali Mohammad Ali Mohammadi, Leila Oryadi Zanjani, Farzam Farahmand

**Affiliations:** ^1^ Department of Mechanical Engineering, Sharif University of Technology, Tehran, Iran; ^2^ Center for Orthopedic Trans-Disciplinary Applied Research, Tehran University of Medical Sciences, Tehran, Iran; ^3^ Orthopedic Surgery Department, Shariati Hospital, Tehran University of Medical Sciences, Tehran, Iran; ^4^ Legal Medicine Research Center, Legal Medicine Organization of Iran, Tehran, Iran

**Keywords:** titanium, tilting suture anchor, biomechanical assessment, human cadaver, ovine statements and declarations

## Abstract

**Introduction:**

A novel titanium tilting suture anchor was designed and fabricated using additive manufacturing. The anchor enjoyed a nonsymmetrical structure to facilitate its insertion procedure through a weight-induced tilt, a saw-teeth penetrating edge to provide a strong initial fixation into cancellous bones of various densities, and an appropriate surface texture to enhance the longterm fixation strength through bone ingrowth.

**Methods:**

Biomechanical tests were performed on 10 ovine and 10 human cadaveric humeri to examine the insertion procedure and assess the initial fixation strength of the anchor, in comparison with a standard screw-type anchor as control.

**Results:**

This study indicated a simple yet reliable insertion procedure for the tilting anchor. All anchors survived after 400 cycles of cyclic loadings and failed in the load-to-failure step. There were no significant differences between the displacements and fixation stiffnesses of the anchors in either group. The ultimate failure load was significantly smaller (p<0.05) for tilting anchors in ovine group (273.7 ± 129.72 N vs. 375.6 ± 106.36 N), but not different in human group (311.8 ± 82.55 N vs. 281.9 ± 88.35). Also, a larger number of tilting anchors were pulled out in ovine group (6 vs. 3) but a smaller number in human group (4 vs. 6).

**Conclusion:**

It was concluded that the biomechanical performance of the designed tilting anchor is comparable with that of the standard screw-type anchors.

## 1. Introduction

Suture anchors are orthopedic implants that are primarily used in minimally invasive surgical procedures to facilitate the reattachment of damaged soft tissues, such as tendons and ligaments, to bones (1). The primary function of anchors is to provide a secure attachment site for the soft tissues by forming a strong and stable fixation into the bone (2). The design of suture anchors has been improved over the years to maximize their functionality by modifying both the material composition and the operational mechanism (2–5).

Regarding the material, suture anchors are commonly fabricated using either biodegradable or metallic materials. Biodegradable anchors can rapidly form a robust biological fixation with the bone following implantation, but they eventually decompose. Nevertheless, there are reports of their inflammatory reactions or complications (6). In contrast, metallic anchors have stronger initial fixation than biodegradable anchors and are considered safer for immune-mediated reactions. Also, the changes in their position can be easily assessed using simple radiographic imaging techniques (7). Titanium exhibits higher biocompatibility than other metallic materials and can activate the human osteoblasts to deposit more calcium-based minerals (8).

Although metallic suture anchors have demonstrated strong initial fixation, previous clinical studies have documented potential complications that may arise later, such as migration, loosening, breakage, and interference with surrounding tissues (9–11). One approach to avoid these complications is providing appropriate conditions for osseointegration and bone ingrowth, hence ensuring the durability of fixation in the long term. New advancements in additive manufacturing technologies, i.e., three-dimensional (3D) printing, have made it possible to create optimized microstructures on the surface of implants that closely match the cancellous bone (12). For example, a study conducted on animals has shown that 3D printed implants exhibit a higher capacity for bone ingrowth than solid implants (13). Based on these insights, several recent studies have developed novel designs of suture anchors that can be fabricated using metallic 3D printing. These designs have been subjected to pull-out tests to evaluate their performance. For instance, Hsieh et al. (9) designed a threadless suture anchor using 3D printing with a rectangular cross section, and Chen et al. (14) utilized 3D printing to develop a novel hybrid suture anchor made of titanium for patients with osteoporosis.

In terms of anchoring mechanism, suture anchors are divided into two main categories: screw-type and impaction-type (7). Screw-type anchors are designed with self-drilling and self-tapping tips to achieve rapid and secure fixation into the bone. They are well-established devices in surgical practice and have excellent clinical outcomes in general. However, their machined-polished surfaces prevent them from fixing biologically into the bone. Consequently, there is a possibility that they may gradually loosen and migrate into the joint space, which can cause serious complications. On the other hand, impaction-type anchors, e.g., press-fit anchors, barbed anchors, winged anchors, and toggling or tilting anchors, are inserted into a predrilled hole within the bone, establishing a mechanical connection with the surrounding walls of the hole (15, 16). The tilting suture anchors are more attractive among impaction-type anchors, due to their secure initial fixation. These anchors are configured to be inserted along their length into the hole and subsequently tilted inside through maneuvers performed by the surgeon', e.g., pulling the suture threads that pass through the anchor's eyelet (6). Some essential advantages of these anchors include the capability of fixation under cortical bone in low-density bones and providing higher resistance to increasing pull-out force by increasing the tilt angle (up to 90°) and the load-bearing area. Moreover, the tilting anchors have the capability of being fabricated using 3D printers, due to their simple geometry and, hence, can enjoy surfaces with favored microstructures for bone ingrowth. The fixation strength of the tilting anchors has been investigated in previous studies using *in vivo* and *in vitro* mechanical testing. For instance, Pietschmann et al. (17) investigated the pull-out strength of BioKnotless RC and UltraSorb suture anchors, and Barber et al. (19) examined the pull-out strength of BioKnotless RC and BioKnotless BR tilting suture anchors.

Considering the advantageous features of the tilting suture anchors on one side and the 3D printed metallic implants on another side, the objective of this study was to develop a titanium tilting suture anchor with improved biomechanical and clinical performance, which could be fabricated using 3D printing. A novel design was introduced for the anchor to fulfill three main functional requirements, including easy surgical procedure, strong initial and long-term fixation, and applicability to cancellous bones of various densities. Subsequently, *in vitro* tests were performed on bone specimens from both animal and human subjects, which exhibited different densities, to assess the implantation procedure and the initial fixation strength, i.e., pull-out strength, of the designed anchor compared with screw-type suture anchors used as the control.

## 2. Method

### 2.1. Anchor design

**Figure 1:**
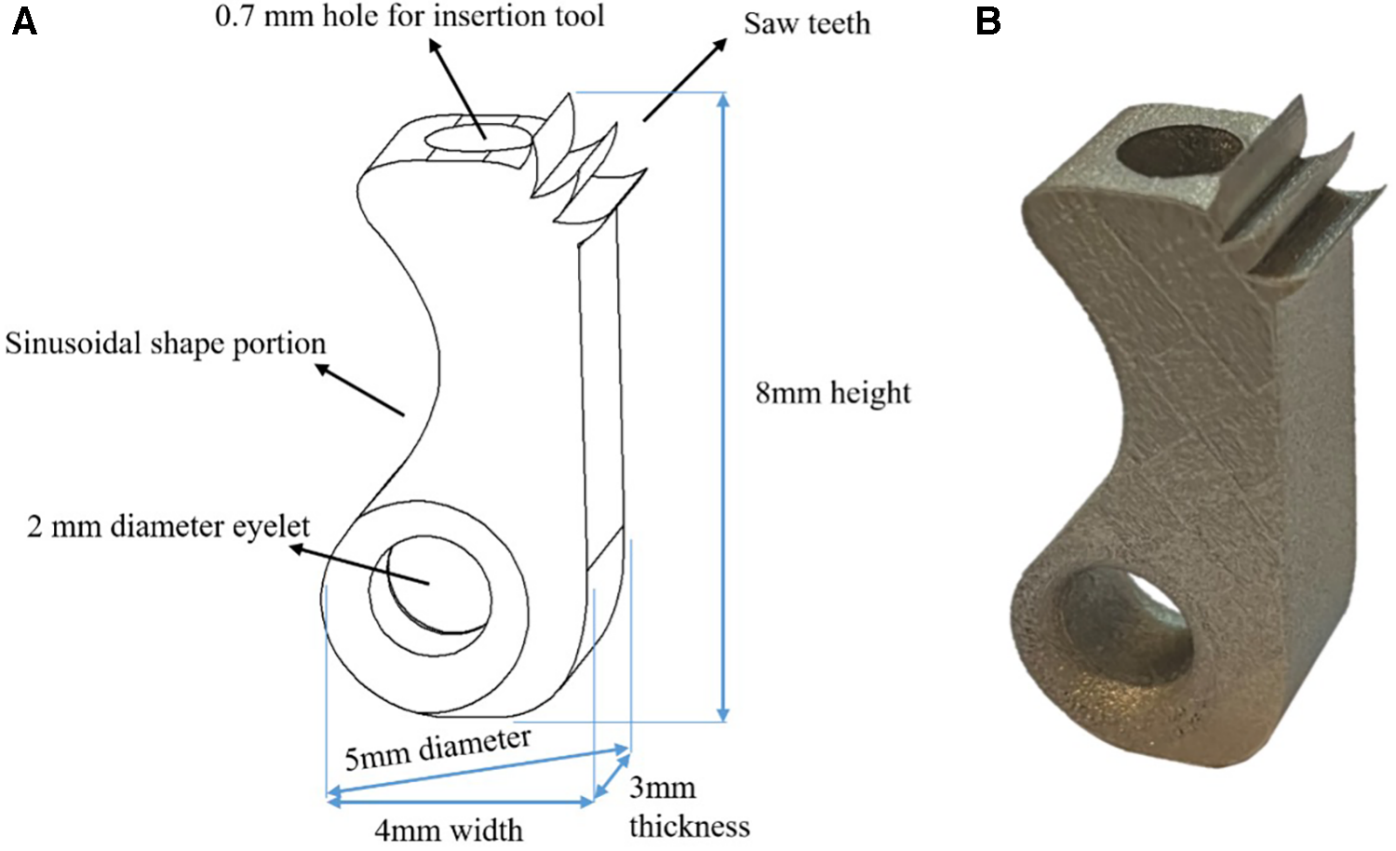
Newly designed tilting suture anchor: (**A**) design schematics and (**B**) 3D printed sample.

**Figure 2:**
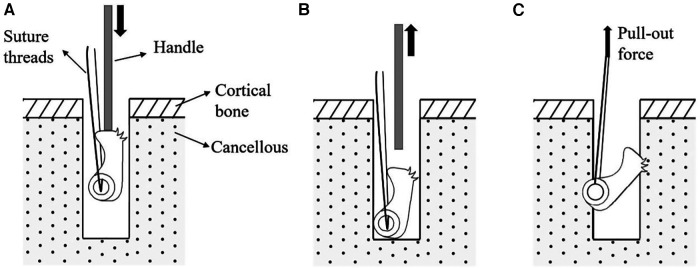
Surgical insertion procedure of the newly designed tilting suture anchor: (**A**) anchor is placed inside the predrilled holes using a handle, (**B**) anchor is released inside the hole, and (**C**) suture is pulled.

A 3D schematic representation of the new tilting suture anchor is shown in Figure 1A. It is 5 mm in diameter and 8 mm in height and has a non-symmetrically located eyelet at a distal corner. The eyelet is large enough to allow for the passage of several suture threads and has round fillets at its edges to preserve the threads from being cut. The anchor has a sinusoidal shape sidewall at the same side of the eyelet to increase the horizontal offset between the eyelet and the anchor's center of mass. This offset is important for facilitating the surgical procedure since it would cause a weight-induced tilt; once the suture is pulled, the anchor would be readily engaged with the walls of the hole, eliminating the need for holding it from sliding during the insertion procedure. In fact, for the insertion of the anchor, a suitable method would involve positioning it in the predrilled hole using a rod-shaped handle, which is connected to the female connector at the anchor's top surface through a slip-fit mechanism, releasing the anchor, and then pulling the suture threads (Figure 2).

The designed anchor is equipped with deep saw teeth on a 0.5 mm radius round surface at its proximal bone engagement edge, which provides an excellent penetration ability into the bone, even that of a high density. This round surface, as well as that of the distal concave portion of the sinusoidal sidewall, also provides a large bone–implant contact area, which helps us to make a strong engagement with the bone, even that of a low density. All these design features are critical to guarantee an initial secure fixation into cancellous bones with different qualities.

Samples of designed tilting anchors were fabricated using a selective laser melting (SLM) machine (M100P, NOURA, Iran) from medical-grade titanium alloy, i.e., Ti6AL4V ELI (Figure 1B). The SLM method enabled applying a proper texture to the 'surface of the anchor with a roughness of Ra = 8 μm to enhance the bone ingrowth into the anchor and improve its long-term fixation strength.

### 2.2. Bone specimens

The experiments of this study were approved by the ethics committee of Tehran University of Medical Science (ethical ID: IR.TUMS.MEDICINE.REC.1400.374). Two groups of bone specimens were used for biomechanical tests. The first group included 10 ovine humeri aged between 4 and 6 months (18, 20–22), which were taken from a local abattoir. Previous studies have reported that the pull-out strength observed in ovine humeri is close to that observed in healthy human humeri (18, 23).

The second group included 10 humeri specimens obtained from human cadavers, with a mean age of 44.6 at the time of death (ranging from 30 to 60 years old). The bone mineral density of the metaphyseal region of the specimens was found using quantitative computed tomography (QCT) with 0.5 mm slice thickness; QCT images were segmented using in-house segmentation software (Avin, Tehran, Iran) to find the HU numbers of the bone voxels, which were then mapped onto the bone mineral density using a linear relationship with the constants obtained from the calibrating phantom (24). The average density of the human humerus specimens was 0.70 g/cm^3^ (standard deviation: 0.17).

### 2.3. Preparation and insertion procedure

All ovine and human specimens were stored at a temperature of −21 °C and were thawed prior to the tests. Subsequently, the specimens were subjected to a thorough removal of soft tissues and sectioned at the proximal third of the shafts. The shafts were embedded in cubic frames composed of dental resin and then fixed into an adjustable fixture with three rotational degrees of freedom.

**Figure 3:**
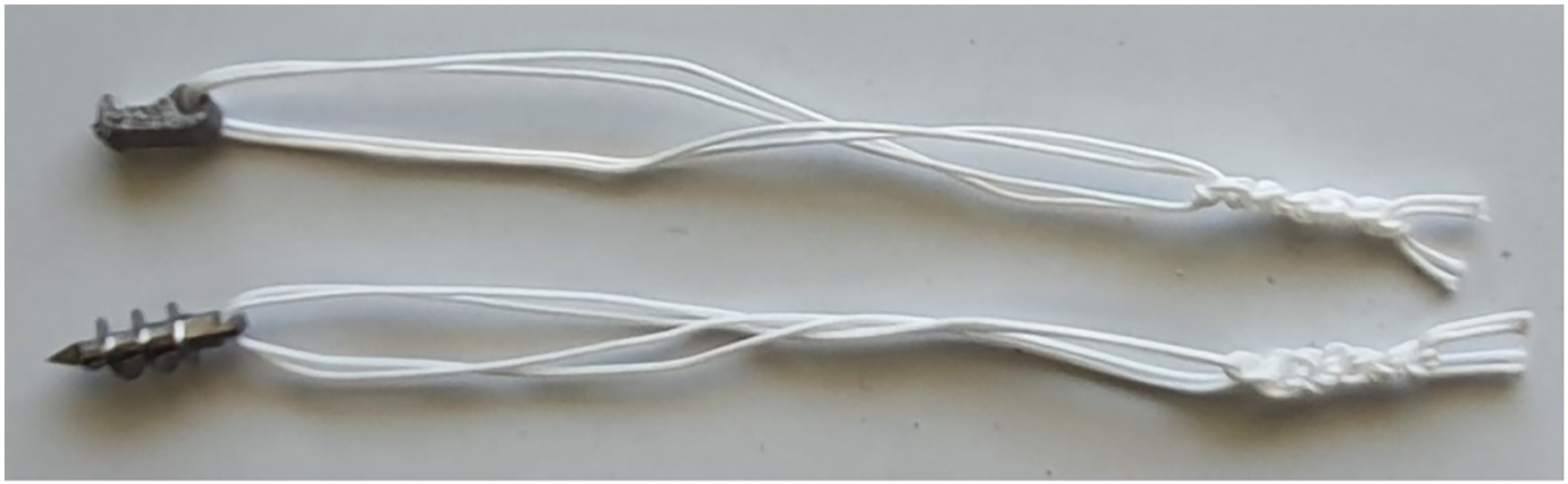
Suture anchors of the novel tilting design (top) and screw-type (bottom) prepared for biomechanical tests.

**Figure 4:**
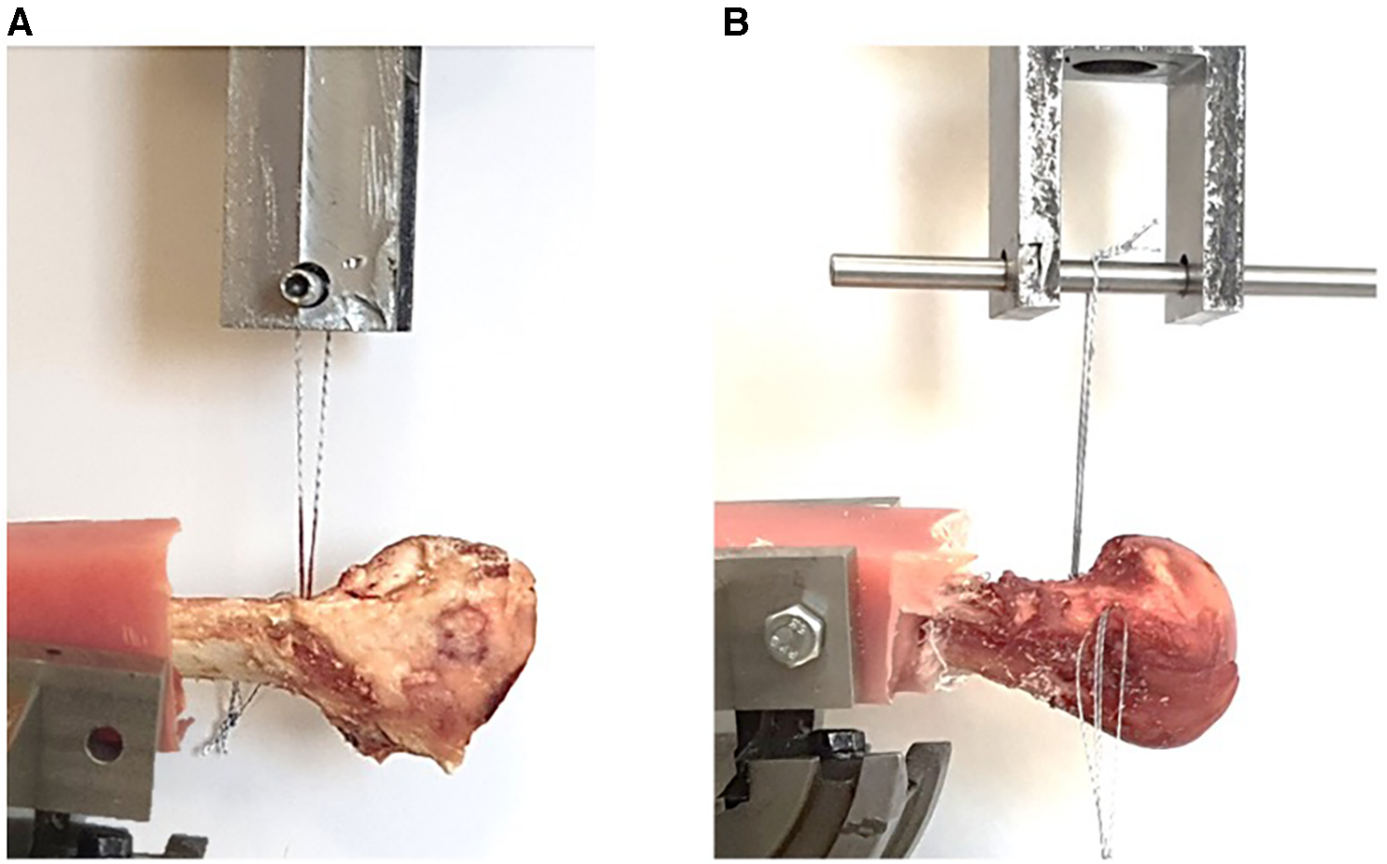
Biomechanical test on (**A**) ovine humerus and (**B**) human humerus specimens.

A commonly used screw-type suture anchor was chosen as a control to be compared with the designed anchor (25). The control anchor was 5 mm Super Revo (ConMed Linvatec, Utica, NY, USA), made from titanium alloy, which has the same diameter as the designed tilting anchor and 14 mm length (Figure 3). A total of 20 tilting suture anchors of the novel design of this study and 20 screw-type suture anchors were prepared for each group of bone specimens. Two strands of #2 Hi-Fi sutures were used for each anchor with the same lengths and similar non-sliding knots (Figure 4). Holes with 5 mm diameter and 15 mm depth were drilled in the metaphyseal parts of the bone specimens perpendicular to their cortical surfaces. The holes were spaced at least 10 mm apart so to ensure that any potential damages incurred during testing would not affect the adjacent holes (3, 26).

Finally, the suture anchors were inserted into the holes. The tilting anchors were placed in the holes along their longitudinal axes using a handle connected to the female connector at the anchor's top surface and then released inside the hole. The screw-type anchors were inserted and fixed in the holes in accordance with the standard insertion procedure and using the instruments recommended by the manufacturer.

### 2.4. Biomechanical tests

Biomechanical tests were performed using a servo-hydraulic testing machine (Amsler HCT 25-400; Zwick/Roell AG, Germany). For each anchor, the suture threads were first looped around a pin attached to the moving jaw of the test machine (Figure 4). The position and orientation of the bone were carefully controlled using the adjustable fixture such that the traction angle of the sutures remained perpendicular to the bone surface. The loading conditions were selected from previous studies (27–29). The anchors were first preloaded to 40 N using the force-control mode of the system to simulate the construct stability testing by surgeons. For tilting anchors, this loading also simulated the surgical maneuver for tilting inside the hole and engaging with the bone. In the second step, 200 cycles of cyclic loading, with a range of 10–50 N, were applied at a frequency of 0.5 Hz. The third step included 200 cycles of cyclic loading with a range of 10–100 N at a frequency of 0.5 Hz. Finally, the anchors were pulled until failure, with a constant velocity of 15 mm/s. The force and displacement data were recorded during tests at a frequency of 25 Hz, and the magnitudes of the following parameters were determined: (1) ultimate failure load, (2) stiffness (determined in the load-to-failure step), and (3) displacements at the end of the first cyclic loading and second cyclic loading, as well as that of the failure point. At the end of the biomechanical tests, radiographs were taken for the test specimens that failed due to the suture breakage in the load-to-failure step to check the ’’configurations of the anchors inside the bone.

### 2.5. Statistical analysis

The Kolmogorov–Smirnov test showed a normal distribution of the data across samples. Two-tailed *t*-tests were used to compare the results. A *p*-value of less than 0.05 indicated a statistically significant difference between results.

## 3. Results

**Figure 5:**
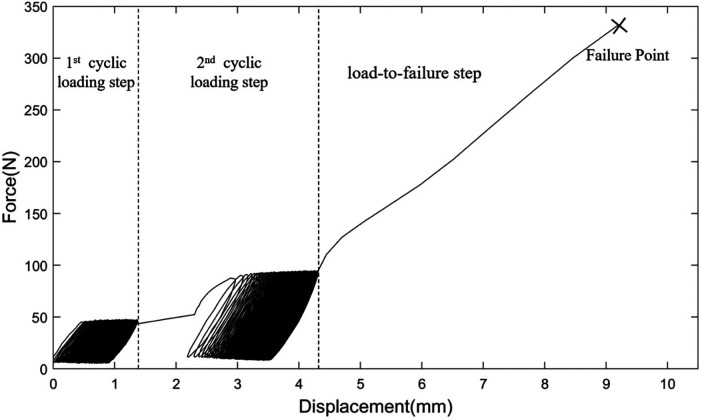
Force–displacement data recorded for a sample of the designed tilting anchor during three steps of the biomechanical test.

**Figure 6:**
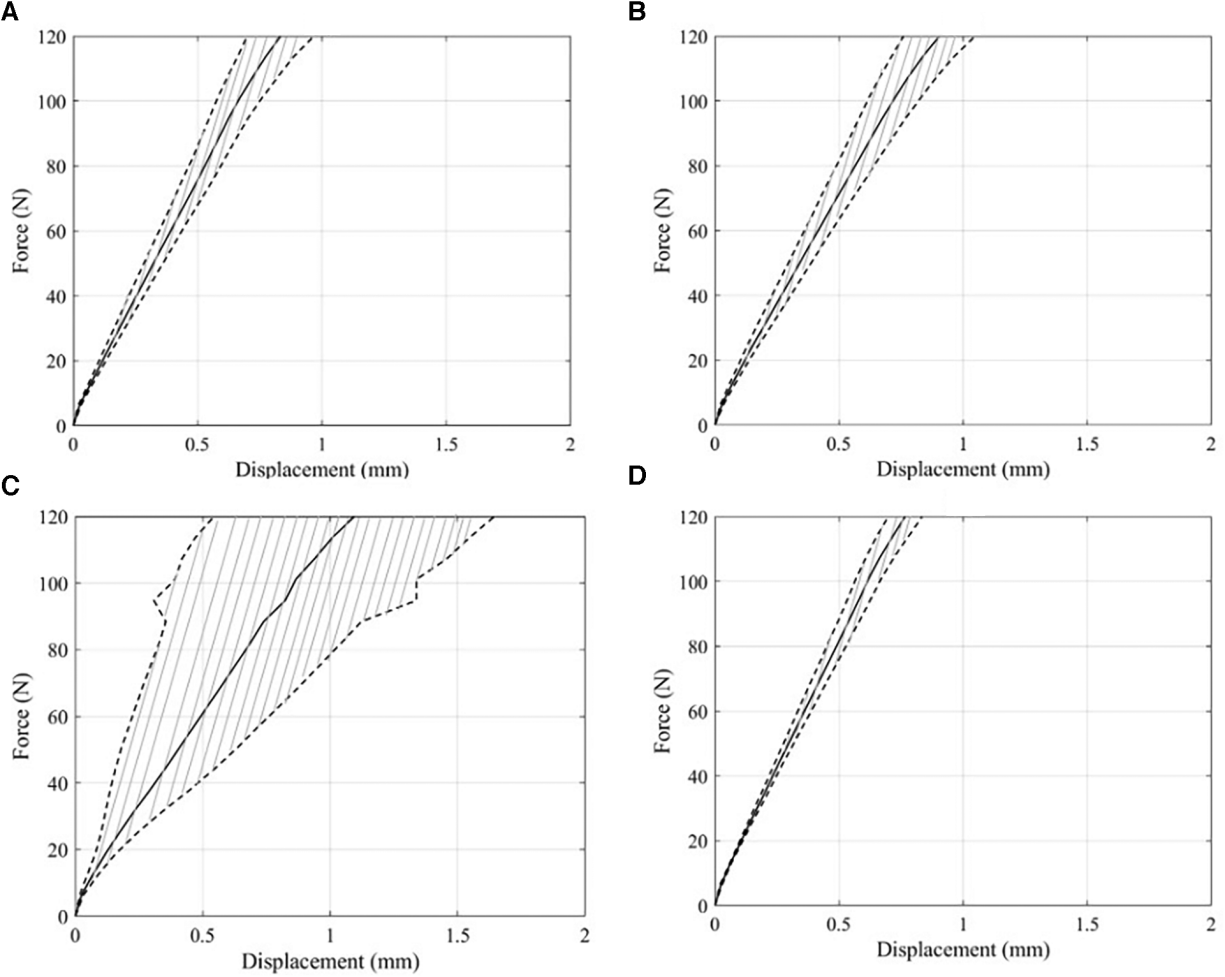
Average and standard deviation of force–displacement curves obtained from load-to-failure steps in pull-out tests. (**A**) Force–displacement curve of tilting bone anchor resulted from pull-out tests on human humeri. (**B**) Result of pull-out tests of screw bone anchors in human humeri. (**C**) Force–displacement curve of tilting bone anchor results from pull-out tests on ovine bones. (**D**) Force–displacement curve of screw bone anchor obtained from pull-out tests on ovine bones.

All the tilting and screw-type anchors were found to be securely affixed to the bone samples, following their relevant insertion procedures. A sample of the recorded data of a tilting anchor during a biomechanical test is shown in Figure 5. The force–displacement diagram included portions from the first cyclic loading step, the second cyclic loading step, and the load-to-failure step, which involved suture breakage. The averaged force–displacement diagrams obtained for each combination of the bone specimens and anchor types during the load-to-failure steps are shown in Figure 6.

**Figure 7:**
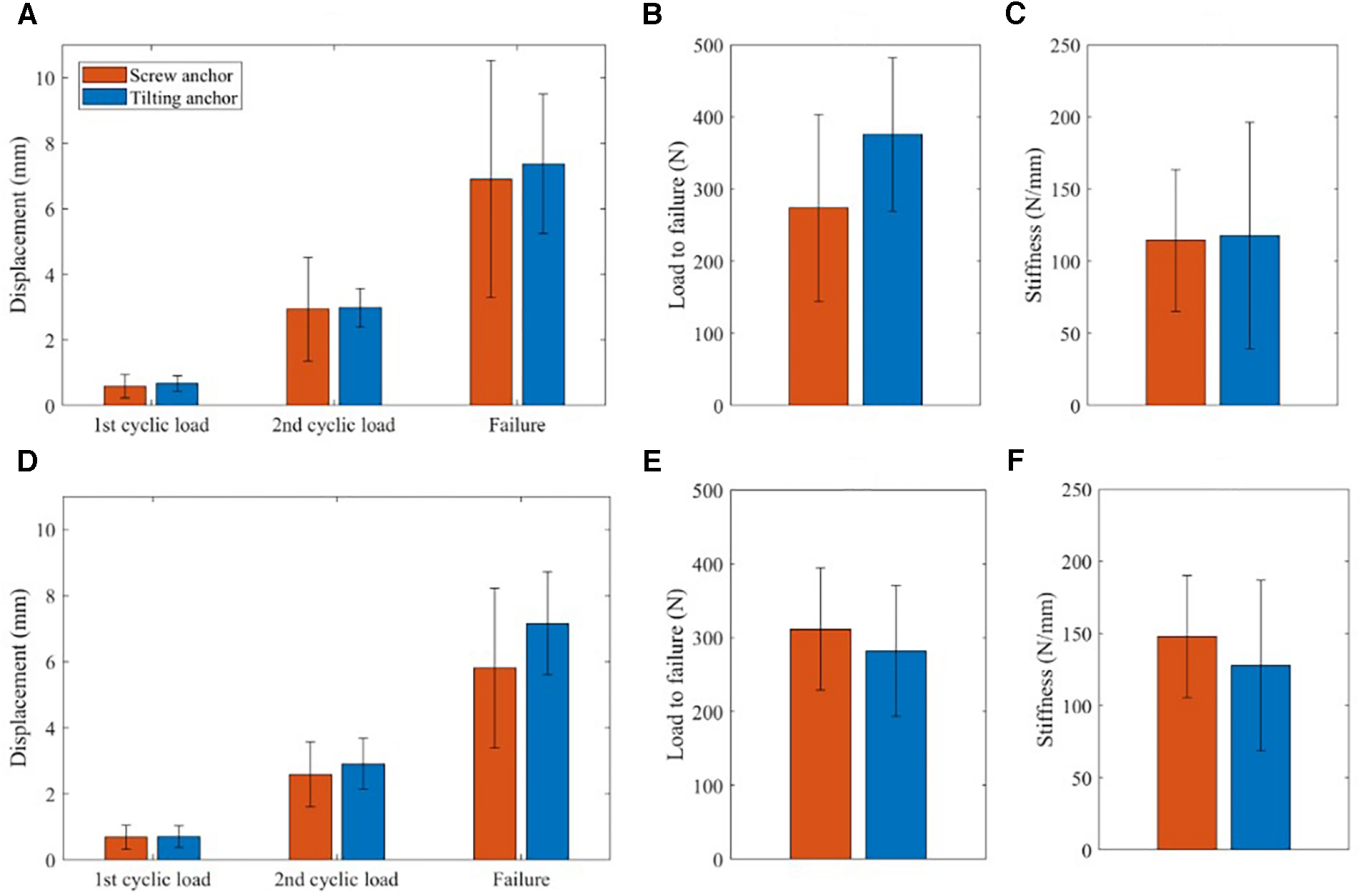
Results of biomechanical tests on ovine and human humerus specimens. Including displacement at the end of the cylic and load to failure steps for ovine group (**A**) and human group (**D**), magnitudes of ultimate loads for ovine group (**B**) and human group (**E**), and stiffness magnitudes, calculated at load to failure step, for ovine group (**C**) and human group (**F**).

The results of the biomechanical tests are summarized in Figure 7. For all specimens of both the ovine and human humerus groups, the anchors survived after 400 cycles of cyclic loading and failed in the load-to-failure step, either due to the suture breakage or the pull out of the anchor from the bone. No significant differences were observed between the displacements of the tilting and screw-type anchors at the end of the cyclic loading and the load-to-failure steps in either of the ovine and human test groups (Figures 7A, D). Similarly, the ultimate failure loads (Figures 7B, E) and fixation stiffnesses (Figures 7C, F) of the two types of anchors were not significantly different. In the ovine group, six tilting anchors and three screw-type anchors experienced pull-out in the load-to-failure step. For the human group, there were four tilting anchors and six screw-type anchors that exhibited pull-out in the load-to-failure step.

**Figure 8:**
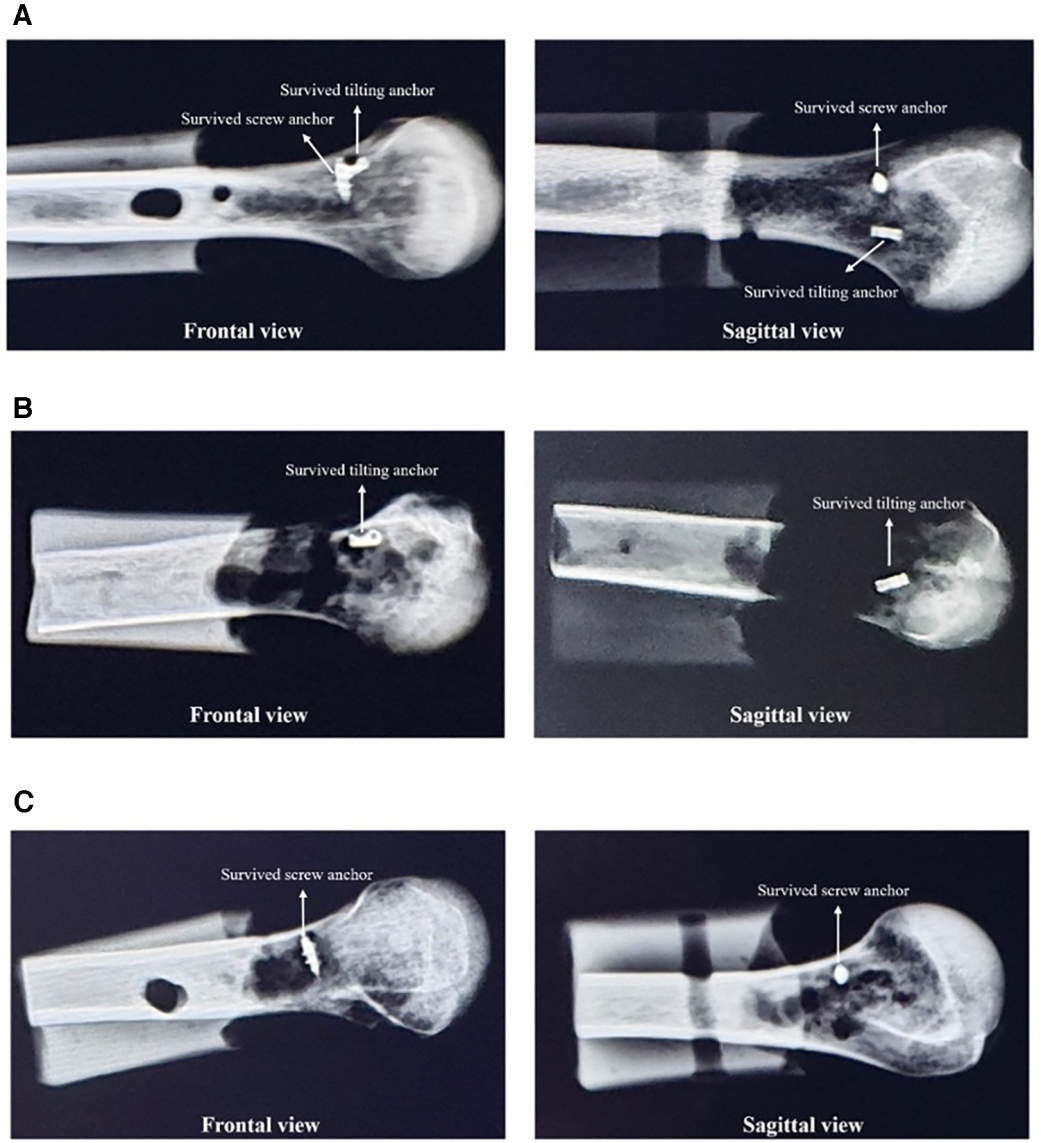
Frontal and sagittal radiographs of sample human humerus specimens taken after the load-to-failure step. (**A**) Human sample #4: suture breakage occurred in both suture anchors. (**B**) Human sample #6: the failure occurred due to suture breakage for the tilting anchor and due to the anchor pull out for the screw-type anchor. (**C**) Human sample #9: the tilting bone anchor was pulled out, but suture breakage happened in the screw suture anchor.

The radiograph of a sample of human humerus specimens, taken after the load-to-failure step, is shown in Figure 8. In the first image, the failure occurred due to the suture breakage, and the tilting anchor remained fixed under the cortex following an approximately 80° tilt. In the second image, the screw anchor was pulled out of the bone, and the tilting bone anchor was fixed horizontally under the cortical bone. Finally, a radiograph was provided to show a state that the tilting bone anchor was pulled out of the bone, and the suture breakage happened for the screw bone anchor.

## 4. Discussion

This study examined the insertion procedure and assessed the initial fixation strength of a novel design of titanium tilting anchor intended to provide a facilitated surgical procedure, a strong initial fixation in cancellous bones of various densities, and a long-term secure fixation through osseointegration and bone ingrowth. The results provided evidence that the designed anchor exhibits an easy and reliable insertion procedure using only a limited number of instruments; the anchor consistently achieved a significant degree of tilting inside the hole, resulting in a reliable mechanical engagement with the bone. This simplified and facilitated procedure is of great importance from a clinical point of view, particularly for minimally invasive surgeries; it makes the designed anchor uniquely advantageous not only over the screw-type anchors, which are hardware-demanding and sensitive to surgical mistakes, but also over the conventional tilting anchors, which require surgical maneuvers involving the use of two hands for holding the insertion handle and simultaneously pulling the suture.

The results of our biomechanical tests also revealed that the designed tilting anchor has a comparable initial fixation with that of the standard screw-type anchor. For both the ovine and human humerus groups, the tilting anchor could tolerate the two steps of cyclic loading, with displacements not significantly larger than those of the screw-type anchor (Figure 7). Moreover, it exhibited similar stiffness, ultimate failure load, and displacement at failure to those of the screw-type anchor.

For the failure mode, however, the results were different for the designed tilting anchor and the screw-type anchor. In the ovine group, with an insignificant difference in ultimate failure loads (273.7 ± 129.72 N vs. 375.6 ± 106.36 N), a larger number of tilting anchors were pulled out compared with the screw-type anchors. Interestingly, this trend was the opposite for the human group. Although the difference between the ultimate failure loads was also insignificant in the human group (311.8 ± 82.55 N vs. 281.9 ± 88.35 N), the tilting anchors experienced fewer pull-outs and a higher number of suture breakages. This observed behavior could potentially be attributed to the significant variations in the densities of the ovine bone specimens (18) and the human bone specimens, as reflected in the substantial standard deviations observed in the corresponding results. It is hypothesized that under conditions of very low-density bones and high loads, the tilting anchor may undergo a substantial tilt angle exceeding 90°, resulting in dislodgment from the hole.

Although the above observation might question the capability of the designed tilting anchor to be used in a wide range of cancellous bone densities, it should be noticed that the failure load remained sufficiently high compared with the physiological loads, which are approximately 50 N in the shoulder and hip (28). Nevertheless, the initial fixation strength of the designed anchor can be improved by some minor modifications, i.e., increasing the height of the anchor from 5 to 7 mm. Considering the mechanism of failure of the anchor in low-density bones, this design modification is expected to enhance its mechanical engagement with the bone at small and medium tilt angles, such that tilt angles larger than 90° are avoided.

The biomechanical results obtained in our study for the tilting and screw-type anchors are comparable with those reported in the literature for different types of anchors. For instance, Pietschmann et al. (29) reported ultimate failure loads in the range of 150–250 N for tilting and 150–225 N for screw-type anchors fixed into human humeri. In another study (18), they found the mean ultimate failure forces of two types of tilting anchors and a similar screw-type anchor of our study as 192, 225, and 187.5 N in the human group and 191, 182.5, and 195 N in the ovine humerus specimens, respectively. Also, Barber et al. (3) reported the means of the ultimate failure loads of two types of tilting anchors fixed into porcine humeri as 242.6 and 268.5 N.

Finally, the selected roughness, applied to the surface of the designed tilting anchor using the SLM fabrication method, is promising for the long-term performance of the anchor. This potential feature of the anchor was not investigated in the current study and is planned to be assessed in future *in vivo* investigations on live animals.

Our study suffered from some limitations, particularly in the number of specimens examined. To enhance the reliability of the results, studies on a larger number of human bone specimens from different ages and genders are required. Also, to provide a deep insight into the results, it is necessary to classify the bone specimens based on their mineral densities and assess the biomechanical performance of the designed anchor in each class separately. Moreover, this study did not investigate the promising features of the designed tilting anchor for long-term fixation strength. *In vivo* examinations on animal models can unveil whether the 3D printed surface texture and improved initial fixation of the anchor provide sufficient requirements for osseointegration and bone ingrowth, guaranteeing that fixation remains strong in the long term. Finally, considering the advantageous capabilities of additive manufacturing over the traditional techniques, the designed tilting anchor can potentially benefit from hybrid materials (30), porous structures (31), and/or special surface treatments (32), the effects of which on the anchor's biomechanical performance will be studied in future investigations.

## 5. Conclusion

Considering the advantages of 3D printing manufacturing and tilting bone anchors, a novel titanium tilting bone anchor was developed to facilitate surgical procedures, a strong initial fixation in cancellous bones of various densities, and a long-term secure fixation through osseointegration and bone ingrowth. The biomechanical tests on ovine and human humeri revealed that the designed tilting bone anchor exhibits an easy yet reliable insertion procedure with minimal instruments and a comparable initial fixation with that of the standard screw-type anchor.

## Data Availability

The raw data supporting the conclusions of this article will be made available by the authors, without undue reservation.
